# An Investigation on the Effects of Dietary Vitamin E on Juvenile Sea Urchin (*Strongylocentrotus intermedius*): Growth, Intestinal Microbiota, Immune Response, and Related Gene Expression

**DOI:** 10.3390/biology12121523

**Published:** 2023-12-14

**Authors:** Min Li, Dan Gou, Panke Gong, Weixiao Di, Lina Wang, Jun Ding, Yaqing Chang, Rantao Zuo

**Affiliations:** 1 Key Laboratory of Mariculture and Stock Enhancement in North China’s Sea (Ministry of Agriculture and Rural Affairs), Dalian Ocean University, Dalian 116023, China; limin40409@163.com (M.L.); yqchang@dlou.edu.cn (Y.C.); 2 Department of Marine Biology, Weihai Ocean Vocational College, Weihai 264300, China

**Keywords:** sea urchin, antioxidant enzymes, inflammation, intestine

## Abstract

**Simple Summary:**

Sea urchin is rare but valuable and considered luxurious sea food due to the gonads with bright color, delicate taste, and abundant nutrition. Since macroalgae have several defects, such as unstable supply, low feed conversion efficiency, and incomplete nutrition, it is essential to formulate feeds suitable for producing sea urchin seeds with higher efficiency. Vitamin E (VE) is an essential nutrient and a lipid-soluble antioxidant for animals. However, no relevant information is available about the requirement of VE and its physiological role in sea urchins. Therefore, this experiment was performed to assess the impacts of dietary VE on growth, intestinal microbiota, immune response, and related gene expression in juvenile *S. intermedius*. It was found that a moderate level of VE (172.5–262.4) can achieve ideal digestive enzyme activities and growth performance, but a relatively higher level of VE (235–302 mg/kg) was beneficial for maintaining the immune and antioxidant capacity of juvenile *S. intermedius* by regulating the expression of inflammation- and immune-related genes and abundance of some bacteria to a healthy state.

**Abstract:**

A 90 d feeding experiment was conducted to investigate the effects of vitamin E (VE) on growth, intestinal microbiota, immune response, and related gene expression of juvenile sea urchin (*Strongylocentrotus intermedius*). Six dry feeds were made to contain graded levels of VE (78, 105, 152, 235, 302, and 390 mg/kg); these were named E78, E105, E152, E235, E302, and E390, respectively. Dry feed E50 and fresh kelp (HD) were used as the control diets. There were six replicates of cages in each dietary group, and each cage held 20 sea urchins with an initial body weight of approximately 1.50 g. Results exhibited that weight gain rate and gonadosomatic index (GSI) of the sea urchins were not significantly affected by dietary VE ranging from 78 to 390 mg/kg. Sea urchins in the dry feed groups showed poorer growth performance, but significantly higher GSI than those in the fresh kelp groups. The pepsin and lipase activities were not significantly promoted by low or moderate VE, but were inhibited by a high level of VE (302–390 mg/kg), while amylase and cellulase activities were significantly increased by low or moderate VE, with the highest values observed in the E105 and E235 groups, respectively. VE addition at a low dosage (105–152 mg/kg) showed inhibitory effects on immune and antioxidant enzyme activities and expression of inflammation-related genes, but showed no beneficial effects at moderate or high dosage (235–390 mg/kg), while a moderate or relatively higher level of VE (235–302 mg/kg) significantly increased the expression of several immune-related genes. The relative abundance of Proteobacteria, Actinobacteria, *Ruegeria,* and *Maliponia* in the intestine of the sea urchins increased with the increase in VE in the dry feeds. On the contrary, the relative abundance of the Firmicutes, Bacteroidetes, *Escherichia-Shigella*, *Bacteroides,* and *Clostridium sensu stricto 1* gradually decreased as VE content increased. These results indicated that a moderate level of VE (172.5–262.4) can achieve ideal digestive enzyme activities and growth performance, but a relatively higher level of VE (235–302 mg/kg) was beneficial for maintaining the immune and antioxidant capacity of juvenile *S. intermedius* by regulating the expression of inflammation- and immune-related genes and abundance of some bacteria to a healthy state.

## 1. Introduction

Sea urchin is rare but valuable and considered a luxurious sea food due to the gonads, characterized by bright color, delicate taste, and abundant nutrition [1]. In recent years, aquaculture has proved to be an effective solution to reduce reliance on wild sea urchins [2]. *Strongylocentrotus intermedius* was imported from Japan to China in 1989 [3]. Since then, a series of studies was conducted on this species, including larval and juvenile production, farming technology, feed manufacture, and disease prevention and control [4,5,6,7,8]. Nowadays, *S. intermedius* has become one of the most important farming species, especially in the coastal areas of northern China [9,10]. Sea urchin seeds are the foundation of massive culture. *Saccharina japonica* and *Undaria pinnatifida* are the natural food during the large-scale larval and juvenile production of *S. intermedius* [10]. However, there are several defects of macroalgae, such as unstable supply, low feed conversion efficiency, and incomplete nutrition. Thus, it is essential to formulate feeds suitable for producing sea urchin seeds with higher efficiency [9,11,12].

Vitamin E (VE) is an essential nutrient and a lipid-soluble antioxidant for animals [13]. Previous studies have shown that diets supplemented with VE increased the immune and antioxidant capacity of aquatic animals, such as rohu (*Labeo rohita*) [14], *Epinephelus Malabaricus* [15], guppy (*Poecilia reticulata*) [16], angel fish (*Pterophyllum scalare*) [17], turbot (*Scophthalmus maximus*) [18], and sea cucumber (*Apostichopus japonicus*) [19]. There have been some studies that aimed to elucidate the beneficial effects of VE on the immune system and antioxidation in a variety of aquatic animals. VE acts as a quencher for reactive oxygen species (ROS) to prevent lipid peroxidation [20]. VE can reduce the activity of cyclooxygenase (COX)-2 and inhibit prostaglandin E_2_ (PGE_2_) synthesis, preventing the occurrence of inflammation and oxidative stress [21,22,23,24]. The intestine not only undertakes the functions of food digestion and nutrient metabolism [25,26,27], but also affects the immune regulation of animals [28]. The role of the intestine in regulating immune response has been accepted and is attracting more and more attention [29]. Huang et al. [30] has found that oxidized fish oil could destroy the intestinal barrier structure of *Ctenopharyngodon Idella* and increase intestinal permeability. Studies on humans and male rats have shown that oxidative stress could destroy the intestinal structure and cause disorders of intestinal microbiota [31,32]. However, no relevant information is available about the requirement of vitamin E and its physiological role in sea urchins.

Therefore, this experiment was performed to assess the impacts of dietary VE on growth, intestinal microbiota, immune response, and related gene expression in juvenile *S. intermedius*. It was expected to quantify the specific VE requirement for juvenile *S. intermedius* based on multiple parameters and improve the understanding of the physiological role of VE in sea urchins.

## 2. Materials and Methods

### 2.1. Feeds and Feeding Procedures

Six dry feeds were formulated by adding graded levels (50, 150, 250, 350, 450, and 550 mg/kg) of VE acetate (purity ≥ 97%, Duly Biotech Co., Ltd., Nanjing, China) according to the estimated VE requirement for aquatic animals [33,34,35]. The final VE contents in the dry feeds were 78, 105, 152, 235, 302, and 390 mg/kg; these were named E78, E105, E152, E235, E302, and E390, respectively. Fresh kelp (*S. japonica*) (HD), the natural food for *S. intermedius,* was used as the control diet. Feed formulation and approximate composition were described in Table 1.

Solid ingredients were crushed to a fine powder, which was passed through a 320 μm mesh. Then, solid ingredients were mixed well by strictly following the feed formulation. Subsequently, fish oil and SL were added and blended evenly again. Finally, water (appropriately 30%) was blended with the mixture, which was used for making pellets. A pellet-making machine (DES-TS1280, Dingrun, China) was used to produce feeds, which were dried at 40 °C. To reduce the oxidation, the dried feeds were packed in sealed bags and stored in a freezer (−20 °C).

The experimental sea urchins were bought from a local farm in Dalian. They were acclimated for 14 days when they arrived at the experimental base. Then, sea urchins of similar size (1.50 ± 0.20 g) were assigned at random to 42 rectangular cages (15×15×35 cm), which were placed in flow-through reinforced plastic tanks (180×100×80 cm). The water flow speed was fixed at appropriately 2.0 L/min. Each cage (replicate) was stocked with 20 individuals. There were six replicates for each dietary treatment. Glass Petri dishes were put at the bottom of all cages to maximally reduce the feed waste. Sea urchins were fed to apparent satiation twice daily (07:00 and 17:00). The residual feeds and feces were removed promptly after each feeding. The following water condition was maintained: water temperature, 14.2–21.8 °C; salinity, 30; pH, 8.0 ± 0.1; and dissolved oxygen, >7.0 mg/L. The whole feeding experiment lasted for 60 days.

### 2.2. Sampling

When the experiment ended, sea urchins in each cage were counted and weighed following 24 h starvation. Subsequently, coelomic fluid was taken from six individuals and then was centrifugated at 4 °C for five minutes (3000 r/min) [35]. After that, the upper fluid was separated and stored at –80 °C. The upper fluid of the coelomic fluid was used for determining related enzyme activities and malondialdehyde content. Finally, all the intestines of the sea urchins were aseptically dissected from each cage. The intestines were divided into three tubes and flash frozen before they were stored at –80 °C. The first tube, with nine intestines, was used for the microbial diversity analysis. The second tube, with six intestines, was used for determining the activities of digestive enzymes. The third tube, with five intestines, was used for the detection of related gene expression.

### 2.3. VE Content Analysis

The VE content was assayed by using the method of Sau et al. [14]. The analysis method can be briefly described as follows. First, samples were saponified to extract VE. After evaporation, the residue was dissolved in methanol. Finally, VE in the dissolved residue was quantified by using reversed-phase high-performance liquid chromatography (Scientific U3000 HPLC, USA). Results were expressed as mg/kg of sample.

### 2.4. Digestive Enzyme and Immune Enzyme Analysis

Crude enzymes were made according to the method of Li et al. [35] First, intestines were homogenized on ice after they were mixed with phosphorate buffer solution (1:9). Then, the homogenate was centrifuged (10,000 g) at 4 °C for 15 min. After that, the crude enzyme extracts at the upper layer were carefully drawn out. Protein contents were assayed according to the method of Bradford [36]. Relevant commercial kits (Nanjing Jiancheng Bioengineering Institute, Nanjing, China) were used to determine the activities of digestive enzymes and immune enzymes.

### 2.5. RNA Extraction and Real-Time Quantitative PCR

The expression of related genes in the intestine was detected with qPCR. The intestines were ground with a tissue grinder (Wuhan Xavier Biotechnology Co., Ltd., Wuhan, China). Trizol universal reagent (DP424, Tiangen, Beijing, China) was used for extracting total RNA. Then, the integrity and the concentration were measured by microspectrophotometer with the Agilent2100 bioanalyzer. After that, cDNA was obtained by using the Prime ScriptTM Real-time PCR Kit (TaKaRa, Beijing, China). Finally, the cDNA templates were diluted by five times before they were used for qPCR. The qPCR was performed by following the instructions of TaKaRa (Dalian, China). LightCycler^®^96 (Roche Group, Basel, Switzerland) was used to perform the qPCR, with primer information displayed in Table 2. The following reaction conditions were used: 95 °C (30 s), 95 °C (5 s) for 40 cycles, 60 °C (32 s). The primer information can be found in Table 3. The relative expression was calculated by using the method of 2^−ΔΔCT^ [9].

### 2.6. Microbial Diversity Analysis

The method of microbial diversity analysis has been described in a previous study by our lab [35]. The related methods can be briefly described as follows.

Total bacterial community DNA was extracted according to the appended protocols of a commercial kit (Omega Bio-tek, Norcross, GA, USA). After that, the V3-V4 region of the bacteria 16S ribosomal RNA gene was amplified by using primer pairs (338F 5’-ACTCCTACGGGAGGCAGCAG-3’ and 806R 5’-GGACTACHVGGGTWTCTAAT-3’). The following PCR reaction regime was used: 95 °C (3 min), 95 °C (30 s) for 27 cycles, 55 °C (30 s), 72 °C (45 s), and a final extension at 72 °C (10 min).

After purified amplicons were obtained, they were sequenced on an Illumina MiSeq platform (Majorbio Biopharm Techonology Co., Ltd., Shanghai, China). Raw fast files were demultiplexed and quality-filtered using QIIME (version 1.17) with the following criteria. UPARSE was used to cluster Operational Taxonomic Units (OTUs) with 97% similarity cutoff. UCHIME was used to identify and remove chimeric sequences.

### 2.7. Data Analysis

Weight growth rate (WGR, %) = (W_f_ − W_i_) × 100/W_i_

Gonadosomatic index (GSI, %) = GM × 100/W

Digestive tract index (DTI, %) = DM × 100/W

where W_i_ and W_f_ are the wet weight of the initial body weight and final body weight of sea urchins; DM, GM, and W are the wet mass of the digestive tract, gonads, and whole body of the selected individuals, respectively.

Data analysis was performed with one-way analysis of variance (ANOVA) following a normal distribution test. If there was significance, Duncan’s multiple range test was used for comparing means between different dietary treatments. *p* < 0.05 was interpreted as the level of significance.

## 3. Results

### 3.1. Growth Performance

WGR increased from 335.1% to 419.9% as dietary VE increased from 78 mg/kg to 235 mg/kg, and then decreased with further increase in VE (*p* > 0.05). WGR of sea urchins in the HD group was higher than that in the dry feed groups, but the significance was only detected between the HD group and E78 group (*p* < 0.05). GSI of sea urchins was not significantly affected by dietary VE in the formulated feed groups (*p* > 0.05). DTI in the E105 was significantly higher than that in the other feed groups (*p* < 0.05). GSI in all dry feed groups was significantly higher than that in the HD (*p* <0.05). On the contrary, DTI in the HD was significantly higher than that in the dry feed groups except for the E105 group (*p* <0.05) (Table 3).

**Figure 1:**
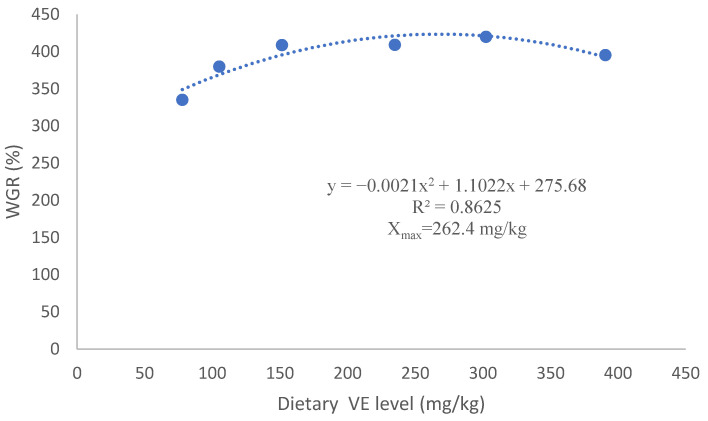
Regression analysis based on WGR.

Based on WGR, the optimal VE requirement was estimated to be 262.4 mg/kg dry feed (Figure 1).

### 3.2. Digestive Enzyme Activities

As dietary VE increased, the activities of pepsin, lipase, amylase, and cellulase first increased and then decreased. The pepsin activity in the HD was significantly higher than that in the E390 (*p* < 0.05), but was comparable to that in the other dry feed groups (*p* > 0.05). Pepsin activity in the E390 was significantly lower than in the other dry feed groups (*p* < 0.05). Sea urchins fed diets with a relatively higher level of VE (302-390 mg/kg) had markedly lower lipase activity than those with a moderate level of VE (152–235 mg/kg) (*p* < 0.05). The lipase activity in the E235 was highest, comparable to that in the low-VE (78–152 mg/kg) groups, but it was significantly higher than that in the other dietary groups (*p* < 0.05). The lipase activity in the HD was significantly lower than that in the E235 (*p* < 0.05), but was comparable to that in the other dry feed groups (*p* > 0.05). The amylase activity in the E235 was highest, comparable to that in the low-VE (105–152 mg/kg) groups, but it was significantly higher than that in the other groups (*p* < 0.05). The cellulase activity in the E105 was highest, comparable to that in the E78 and HD groups (*p* > 0.05), but it was significantly higher than that in the other groups (*p* < 0.05) (Table 4).

**Figure 2:**
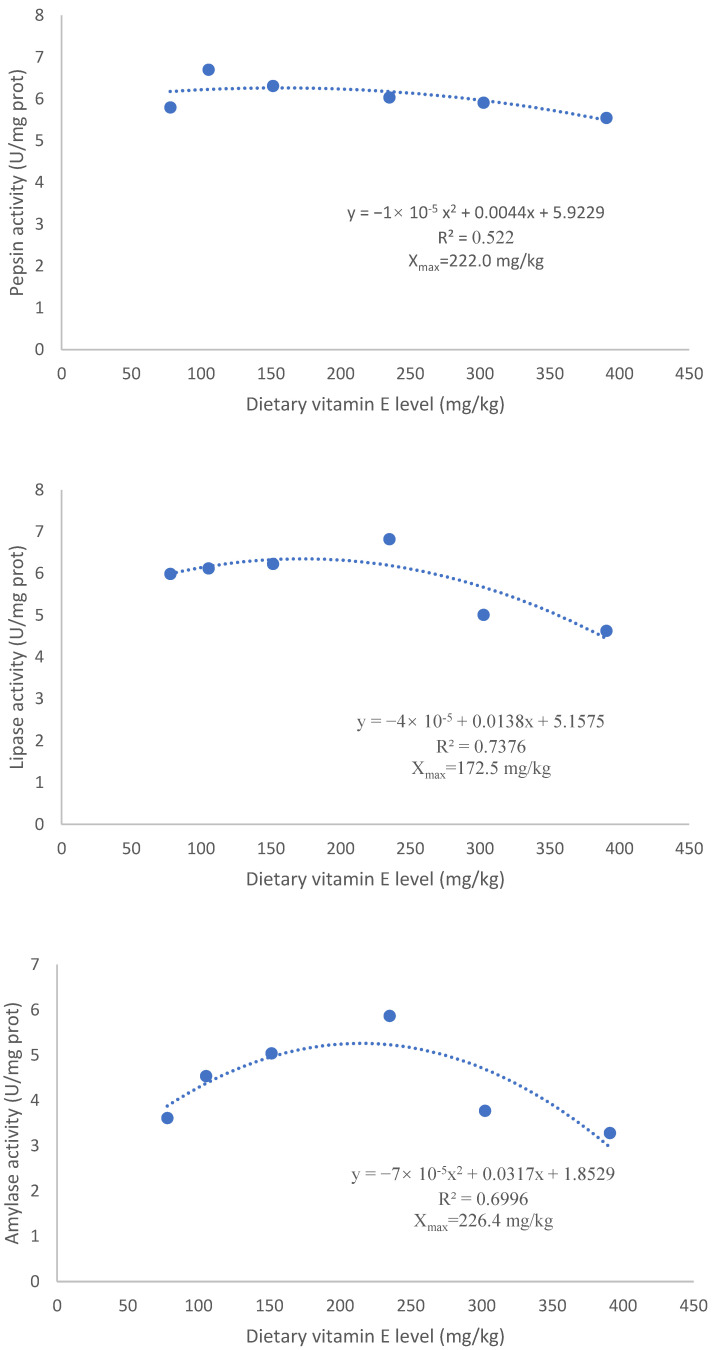
Regression analysis based on digestive enzyme activities.

Based on pepsin activity, lipase activity, and amylase activity, the optimal VE requirement was estimated to be 220.0 mg/kg, 172.5 mg/kg, and 226.4 mg/kg, respectively (Figure 2).

### 3.3. Immune- and Antioxidation-Related Parameters

The activities of immune enzymes, lysozyme (LYZ), alkaline phosphatase (AKP), and acid phosphatase (ACP), in the HD were significantly lower than those in the dry feed groups (*p* < 0.05). The activities of selected immune enzymes and superoxide dismutase (SOD) were decreased by the supplementation of VE (105–390 mg/kg) to different extents. However, no significant differences were detected in the activities of immune enzymes and SOD among the VE supplementation groups (*p* > 0.05). The activities of antioxidant enzymes, catalase (CAT), glutathione peroxidase (GPX), and glutathione S-transferase (GST), first decreased significantly as dietary VE increased from 78 mg/kg to 152 mg/kg, and then increased significantly as dietary VE further increased (*p* < 0.05). The MDA content in HD was significantly lower than that in the dry feed groups except for E105 (*p* < 0.05). Among dry feed groups, MDA content in the E235 was markedly higher than that in the other groups (*p* < 0.05) (Table 5).

### 3.4. Immune-Related Gene Expression

**Figure 3:**
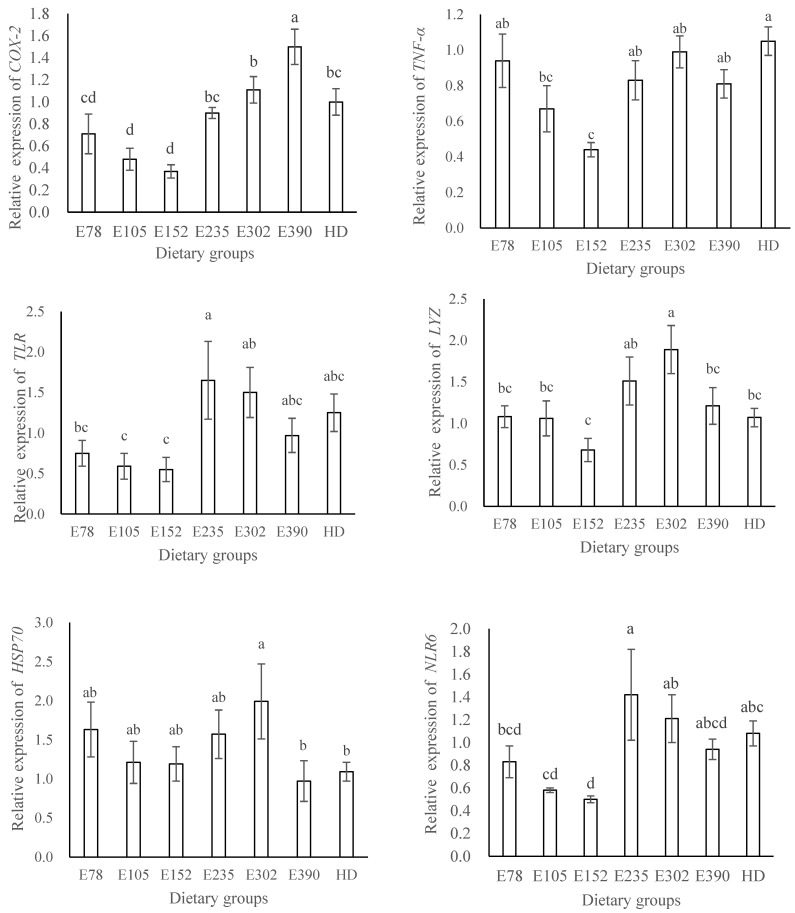

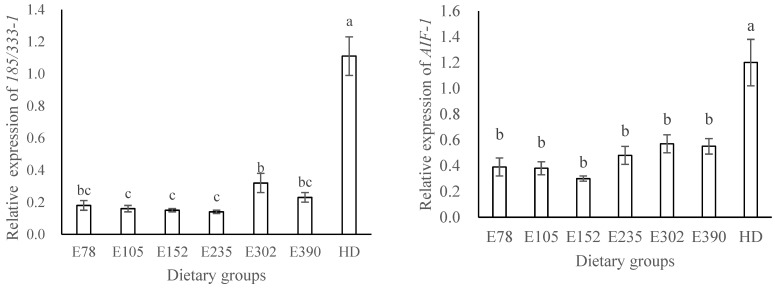
Effects of dietary vitamin E on the intestinal immune-related gene expression of sea urchin (*Strongylocentrotus intermedius*) (mean ± SEM, n = 6). Bars with different letters indicate that the means are significantly different at *p* < 0.05. HD: fresh kelp.

The expression of *COX-2* and *TNF-α* first decreased as dietary VE increased from 78 mg/kg to 152 mg/kg, and then significantly increased with VE further increasing to 390 mg/kg (*p* < 0.05). The expression of *TLR*, *LYZ*, *NLR6,* and *185/333-1* showed no significant differences with VE ranging from 78 mg/kg to 152 mg/kg (*p* > 0.05), and then significantly increased as VE increased to 235 mg/kg or 302 mg/kg (*p* < 0.05). Sea urchins in the group with the highest VE (390 mg/kg) showed markedly lower expression of *TLR*, *LYZ*, HSP70, and *NLR6* than those with a moderate or relatively higher level of VE (235–302 mg/kg). The expression of *COX-2*, *TNF-α*, *TLR*, *LYZ*, *HSP70,* and *NLR6* in HD was comparable to that in E78 (*p* > 0.05). The expression of *185/333-1* and AIF-1 in HD was significantly higher than that in the dry feed groups (*p* < 0.05) (Figure 3).

### 3.5. Intestinal Microbiota

**Figure 4:**
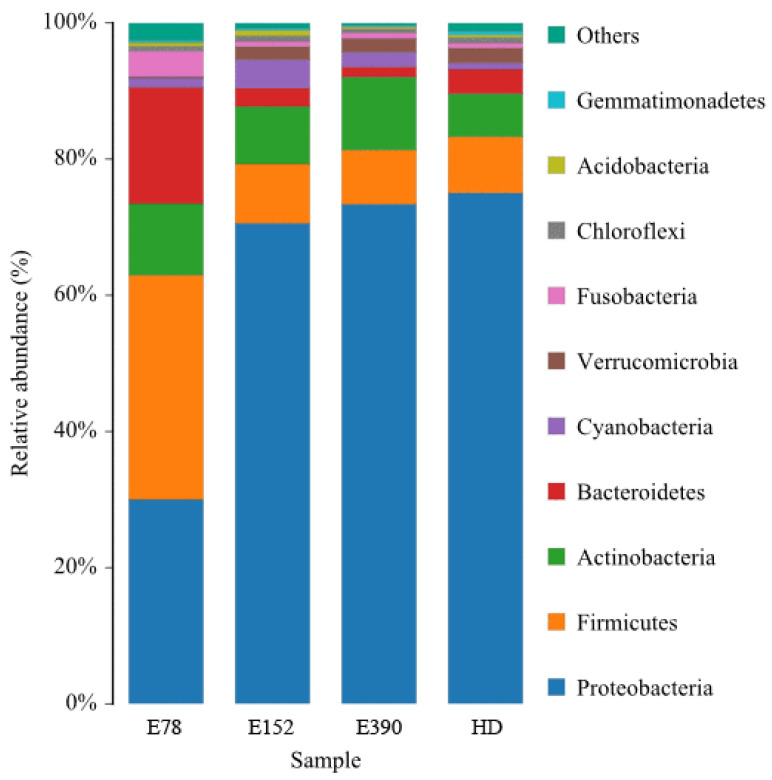
Relative abundance of the intestinal microbiota at the phylum level of sea urchin (*Strongylocentrotus intermedius*) fed different diets (standardized to *18S* rRNA) (mean ± SEM, n = 6). HD: fresh kelp.

*Proteobacteria*, *Firmicutes*, *Actinobacteria,* and *Bacteroidetes* were the main phyla, with their abundance exceeding 90% of the intestinal microbiota in all dietary groups (Figure 4). The relative abundance of *Proteobacteria* significantly increased with increasing dietary VE (*p* < 0.05). The *Proteobacteria* abundance in E78 was significantly lower than that in the other groups (*p* < 0.05). On the contrary, the relative abundance of *Firmicutes* and *Bacteroidetes* significantly decreased as dietary VE increased (*p* < 0.05). *Firmicutes* and *Bacteroidetes* in E78 showed significantly higher abundance than those in the other groups (*p* < 0.05). No significant differences were detected in the relative abundance of *Actinobacteria* among dry feed groups (*p* > 0.05). The relative abundance of *Actinobacteria* in E390 was significantly higher than that in HD (*p* < 0.05) (Table 6).

**Figure 5:**
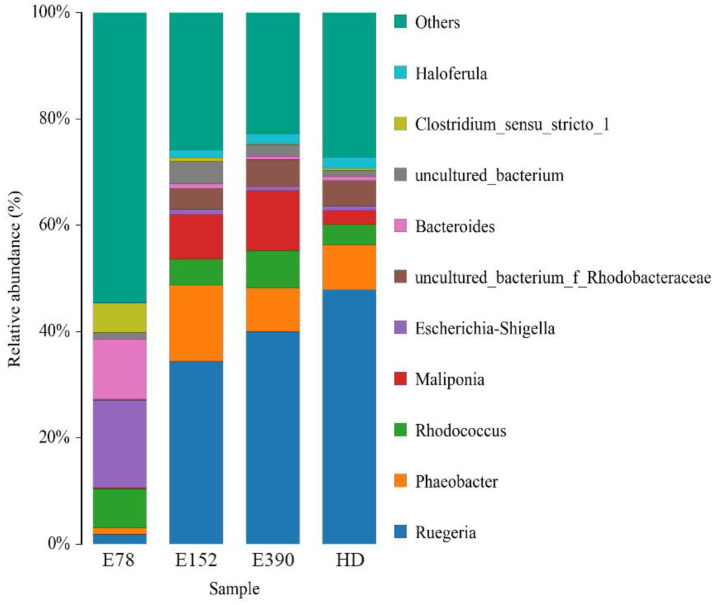
Relative abundance of the intestinal microbiota at the genus level of sea urchin (*Strongylocentrotus intermedius*) fed different diets (standardized to *18S* rRNA) (mean ± SEM, n = 6). HD: fresh kelp.

As for genus, Ruegeria, Phaeobacter, Rhodococcus, Maliponia, Escherichia-Shigella, and Bacteroides were the main intestinal bacteria in all dietary groups (Figure 5). Ruegeria, Phaeobacter, Rhodococcus, Maliponia, Escherichia-Shigella, Bacteroides, and Clostridium sensu stricto 1 were significantly affected by VE in the diets. The relative abundance of Ruegeria and Maliponia significantly increased with increasing dietary VE. The relative abundance of Ruegeria in the E390 group was comparable to that in HD (*p* > 0.05), but was significantly higher than that in E78 and E152 (*p* < 0.05). The Maliponia in E390 showed slightly higher abundance than that in E152 (*p* > 0.05), but was significantly higher than that in the E78 and HD groups (*p* < 0.05). The relative abundance of Phaeobacter increased first and then decreased with increasing dietary VE. The highest abundance of Phaeobacter was observed in the E152 group, significantly higher than that in the other groups (*p* < 0.05). The relative abundance of Escherichia-Shigella, Bacteroides, and Clostridium sensu stricto 1 significantly decreased with increasing dietary VE. Escherichia-Shigella, Bacteroides, and Clostridium sensu stricto 1 in E78 showed significantly higher abundance than those in other groups (*p* < 0.05) (Table 7).

## 4. Discussion

In this study, the WGR of sea urchins fed kelp was significantly higher than that in the dry feed groups. This was in accordance with the findings on this and other sea urchin species [9,14,44]. Kelp is rich in cellulose and mucus, which may be beneficial for protecting the fragile intestine and improving the digestibility of juvenile sea urchins [9,44]. Furthermore, the absolute digestive tract weight of sea urchins fed fresh kelp was significantly higher than that in the other dietary groups. This further proved that fresh kelp was beneficial for the intestinal development of sea urchins. It was previously proved that the addition of VE within a certain dose range can significantly increase the intestinal villi height and mucosal thickness of catfish (*Silurus asotus*), thereby improving intestinal function [45]. Compared to the low-VE groups (78–105 mg/kg), the WGR of sea urchins in the moderate or relatively higher VE groups (152–302 mg/kg) showed an obvious increase, but declined in the highest VE group (390 mg/kg). The estimated VE requirement (262.4 mg/kg) based on the WGR regression model was higher than that for sea cucumber (*Apostichopus japonicus*) (165.2–187.2 mg/kg) [8], that for medaka (*Oryzias latipes*) (121.3 mg/kg) in both the juvenile and adult stages [46], that for Caspian trout (*Squaliobarbus ourriculus*) (78.73–82.16 mg/kg) [47], and that for spotted bass (*Pomoxis nigromacufatus*) (48.2–55.7 mg/kg) [48], respectively. Excessive vitamin E leads to decreased growth performance of grass shrimp (*Penaeus monodon*) [49], and poor growth, hepatotoxicity, and death of keeling (*Tor tambra*) fingerlings [50]. Digestive enzymes are critical for maintaining nutrient digestion and controlling growth performance of aquatic animals [51]. In this study, the activities of digestive enzymes were increased by the appropriate amount of VE (235 mg/kg), but these beneficial effects were removed by an overdose of VE. This was consistent with the findings on channel catfish (*Ictalurus punctatus*) [44] and sea cucumber [52], which suggests that moderate amounts of VE can promote digestive enzyme activity, while excessive VE may inhibit its activity [53].

In this study, the sea urchins fed kelp had significantly lower GSI than those fed dry feeds. This was consistent with the results of some previous studies [9,10,54,55]. Compared to dry feeds, kelp had relatively lower contents of proteins and lipids. It was found that the GSI of juvenile *S. intermedius* increased with the increasing dietary protein concentration [55]. Furthermore, insufficient lipid intake decreased gonad production of sea urchin *Lytechinus variegatus* [56]. Dietary lipid at a level of 90 g/kg achieved the maximum GSI in both male and female *Onychostoma macrolepis* broodstock [57]. A relatively higher lipid level (180 g/kg) in the diets significantly promoted gonad development of female Chinese sturgeon (*Acipenser sinensis*) [58]). There have been many studies that have reported the beneficial effects of VE on the growth and development of red crayfish (*Cherax quadricarinatus*) [59], Nile tilapia (*Oreochromis niloticus*) [60], and pandani (*Pseudotropheus socolofi*) [40].

SOD, CAT, and GPX can be used as “guards” protecting the organisms from the destruction of free radicals [61,62,63,64]. In the present study, the antioxidant enzyme activities were reduced by a low or moderate content of VE (105–152 mg/kg), but were increased as VE content reached levels equal to or above 235 mg/kg. This indicated that VE could preferentially react with free radicals [65], which resulted in a low state of “guards”. However, excessive VE can be subject to lipid peroxidation and caused oxidative stress, reflected by induced inflammation and elevated antioxidant enzyme activities [66,67]. Oxidative stress can induce an inflammation reaction or be induced by inflammation [68,69]. Cyclooxygenase 2 (*COX-2*) can catalyze ARA to prostaglandin E_2_ (PGE_2_). PGE_2_ participates in the activation of downstream inflammatory cytokines [70,71,72]. Song et al. [73] found that oxidative stress can induce inflammation through the hepatic inflammatory signaling pathway. In this study, the expression of inflammation-related genes, such as *COX-2*, *TLR*, *TNF-α,* and *AIF-1*, in the intestine of *S. intermedius* showed a parallel changing tendency to the activities of antioxidant enzymes as dietary VE increased. In this study, the MDA content in the HD group was lower than that in the dry feed groups. The antioxidant substances present in the fresh kelp can resist oxidative stress and protect cells from the hazards of free radicals and other harmful substances. It was previously found that low-molecular-weight fucoidan from the kelp *Laminaria japonica* exerted antioxidant and anticoagulant effects [74]. Fucoidan and kelp sulfated polysaccharide are both polysaccharides with antioxidant properties. Compared with fucoidan, kelp sulfated polysaccharide has much stronger antioxidant activity [75]. Furthermore, the natural fucoxanthin from kelp has high antioxidant and functional properties, and can be used as an alternative raw material for commercial fucoxanthin production [76]. Polyphenolic extracts from marine brown macroalgae were shown to effectively remove oxidants from cells and cellular systems [77].

In the present study, the relative abundance of Proteobacteria, Actinobacteria, *Ruegeria*, *Phaeobacter,* and *Maliponia* in the intestine of the sea urchins increased as dietary vitamin E increased. Porsby et al. [78] found that some bacteria in the genus of *Ruegeria* showed certain antibacterial properties in turbot (*Scophthalmus maximus*). *Phaeobacter* contains some bacteria with obvious bacteriostatic effects [78]. Therefore, it could be the relatively higher abundance of *Ruegeria* and *Phaeobacter* in the intestine that accounted for the beneficial effects of VE and fresh kelp on nonspecific immunity of *S. intermedius*. In this study, the relative abundance of the Firmicutes, Bacteroidetes, *Escherichia-Shigella*, *Bacteroides,* and *Clostridium sensu stricto 1* gradually decreased with increasing VE content. Firmicutes is involved in the absorption of lipids [79]. VE has been found to sooth the abnormal deposition of lipids in the liver by decreasing lipid absorption and increasing lipid oxidation in several fish species. Since *Bacteroides* has a strong capacity of digesting fibers [80], it was postulated that sea urchins are prone to use less fibers for energy substances with the increase in VE in the formulated feeds. Consistently, the cellulase activities decreased significantly as dietary VE increased. *Bacteroides* is abundant in organisms with digestive tract diseases [81]. And *Clostridium sensu stricto 1* also causes intestinal diseases [82]. Therefore, increasing VE could exert its beneficial effects on immune response by decreasing the abundance of some bad bacteria.

## 5. Conclusions

In conclusion, the optimal VE requirement was estimated to be 262.4 mg/kg based on WGR, and 172.5–226.4 mg/kg based on digestive enzyme activities. VE addition at a low dosage (105–152 mg/kg) showed inhibitory effects on immune and antioxidant enzyme activities and expression of inflammation-related genes (*COX-2* and *TNF-α*), but showed no beneficial effects at moderate or high dosage (235–390 mg/kg), while a moderate or relatively higher level of VE (235–302 mg/kg) significantly increased the expression of several immune-related genes, such as *TLR*, *LYZ*, *NLR6,* and *185/333-1*. The relative abundance of Proteobacteria, Actinobacteria, *Ruegeria,* and *Maliponia* in the intestine of the sea urchins increased with the increase in VE in the dry feeds. On the contrary, the relative abundance of the Firmicutes, Bacteroidetes, *Escherichia-Shigella*, *Bacteroides,* and *Clostridium sensu stricto 1* gradually decreased as VE content increased. These results indicated that a moderate level of VE (172.5–262.4) can achieve ideal digestive enzyme activities and growth performance, but a relatively higher level of VE (235–302 mg/kg) was beneficial for maintaining the immune and antioxidant capacity of juvenile *S. intermedius* by regulating the expression of inflammation- and immune-related genes and abundance of some bacteria to a healthy state. 

## Figures and Tables

**Table 1:** Formulation and approximate composition of the experimental feeds (% dry diet).

Ingredients (%)	Experimental Feeds					
	E78	E105	E152	E235	E302	E390
Fish meal	9.00	9.00	9.00	9.00	9.00	9.00
Soybean meal ^a^	17.00	17.00	17.00	17.00	17.00	17.00
Seaweed meal	2.00	2.00	2.00	2.00	2.00	2.00
Ruppiaceae	8.00	8.00	8.00	8.00	8.00	8.00
Wheat bran ^b^	11.20	11.20	11.20	11.20	11.20	11.20
Wheat meal ^c^	29.50	29.49	29.48	29.47	29.46	29.45
Shell powder	6.00	6.00	6.00	6.00	6.00	6.00
Gelatin	5.00	5.00	5.00	5.00	5.00	5.00
Vitamin E acetate	0.00	0.01	0.02	0.03	0.04	0.05
Mineral premix ^d^	2.00	2.00	2.00	2.00	2.00	2.00
Calcium propionate	0.10	0.10	0.10	0.10	0.10	0.10
Betaine	0.10	0.10	0.10	0.10	0.10	0.10
Glycine	0.10	0.10	0.10	0.10	0.10	0.10
Fish oil	8.00	8.00	8.00	8.00	8.00	8.00
Soybean lecithin	2.00	2.00	2.00	2.00	2.00	2.00
Approximate analysis						
Crude lipid	12.24	12.22	12.22	12.21	12.20	12.23
Crude protein	25.79	25.84	25.87	25.84	25.85	25.82
Vitamin E (mg/kg)	77.86	105.27	151.55	235.01	302.43	390.67

^a^ Soybean meal: crude protein 49.4%, crude lipid 0.9%; ^b^ Wheat bran: crude protein 15.8%, crude lipid 4.0%; ^c^ Wheat meal: crude protein 16.4%, crude lipid 1.0%. ^d^ Mineral premix (mg or g kg^−1^ diet): CuSO_4_·5H_2_O, 10 mg; Na_2_SeO_3_ (1%), 25 mg; ZnSO_4_·H_2_O, 50 mg; CoCl_2_·6H_2_O (1%), 50 mg; MnSO_4_·H_2_O, 60 mg; FeSO_4_·H_2_O, 80 mg; Ca (IO_3_)_2_, 180 mg; MgSO_4_·7H_2_O, 1200 mg; zeolite, 18.35 g.

**Table 2:** Real-time quantitative PCR primers used in the present study ^1^.

Gene	Sequence (5′–3′)	Reference
*18S*	F: GTTCGAAGGCGATCAGATAC R: CTGTCAATCCTCACTGTGTC	Zhou et al. [37].
*COX-2*	F: GAGGTGGATAACCGATTGA R: AGCATTGCCCATAGAACAG	MH516324
*NLR-6*	F: GTTCAGGGAGAGGCAGG R: CATGGGCGAGTGGTCAC	Chen et al. [38]
*TLR*	F: TCAAATGGAGCCCGTATGTAGAG R: CTAATGTCCCCTGCTCTGCCA	Wang et al. [39]
*GPX*	F: CGAGTTTGAGAAGCGTGGTG R: GGATCAGCTATGATTGGGTATGG	Ding et al. [40]
*GST*	F: CTCGGAGATTCGCTCACCA R: GCTGGCTGGAGAAATGAACAA	Ding et al. [41]
*HSP70*	F: ACACTCATCTCGGAGGAG R: CTTTCTTATGCTTTCGCTTGA	Bai et al. [41]
*185/333-1*	F: GCTCTTGCTATCTCGGCTCAC R: AAGCGACCTTGTCCTCTCTCTCT	Wang et al. [42]
*LYZ*	F: GAGACGGTACAGGGCTACA R: CGGGCAAAATCCTCACAAG	Ji et al. [43]
*AIF-1*	F: TCGAACGTGCAAGGTGGCAAG R: CGTCATTGTCATCGAGGTCTCCAC	MH516330
*TNF-α*	F: GCTGTAACGGCGTTCGTCTCC R: TGGTGTACTTGTGCTGGTTGTTGG	MH516331

^1^ Cyclooxygenase-2 (COX-2), nucleotide-binding domain leucine-rich repeat 6 (NLR6), toll-like receptor (TLR), glutathione peroxidase (GPX), glutathione S transferase (GST), heat shock protein 70 (HSP70), lysozyme (LYZ), allograft inflammatory factor-1 (AIF-1), tumor necrosis factor α (TNF-α).

**Table 3:** Growth performance of sea urchin (*Strongylocentrotus intermedius*) fed different diets (mean ± SEM, n = 6) ^1^.

Index	Dietary Treatments						
	E78	E105	E152	E235	E302	E390	HD
W_i_ (g)	3.66 ± 0.09	3.66 ± 0.25	3.97 ± 0.18	3.56 ± 0.15	3.90 ± 0.60	3.53 ± 0.09	3.98 ± 0.27
W_f_ (g)	19.83 ± 0.80 ^b^	20.86 ± 1.35 ^ab^	21.57 ± 1.22 ^ab^	20.10 ± 1.27 ^ab^	21.78 ± 1.21 ^ab^	22.18 ± 1.10 ^ab^	24.17 ± 1.87 ^a^
WGR (%)	335.1 ± 26.7 ^b^	380.1 ± 38.6 ^ab^	408.8 ± 38.0 ^ab^	409.1 ± 35.7 ^ab^	419.9 ± 34.0 ^ab^	395.6 ± 14.6 ^ab^	475.1 ± 54.9 ^a^
GM (g)	2.97 ± 0.11 ^ab^	3.17 ± 0.37 ^a^	3.28 ± 0.20 ^a^	3.02 ± 0.25 ^ab^	3.26 ± 0.36 ^a^	2.64 ± 0.72 ^ab^	2.24 ± 0.25 ^b^
GSI (%)	15.21 ± 0.36 ^a^	14.42 ± 0.90 ^a^	14.61 ± 0.61 ^a^	15.36 ± 0.37 ^a^	15.83 ± 0.56 ^a^	14.41 ± 0.26 ^a^	8.67 ± 0.56 ^b^
DM (g)	1.15 ± 0.08 ^b^	1.39 ± 0.12 ^b^	1.21 ± 0.13 ^b^	1.11 ± 0.12 ^b^	1.32 ± 0.17 ^b^	1.12 ± 0.09 ^b^	1.81 ± 0.22 ^a^
DTI (%)	5.80 ± 0.34 ^bc^	6.70 ± 0.18 ^a^	6.31 ± 0.33 ^bc^	6.04 ± 0.44 ^bc^	5.91 ± 0.30 ^bc^	5.55 ± 0.71 ^c^	7.62 ± 0.44 ^a^

^1^ Mean values with different superscript letters within the same row are significantly different at *p* < 0.05. Fresh kelp (HD), initial body weight (W_i_), final body weight (W_f_), weight growth rate (WGR), gonad mass (GM), gonadosomatic index (GSI), digestive tract mass (DM), digestive tract index (DTI).

**Table 4:** Digestive enzymes in the digestive tract of sea urchin (*Strongylocentrotus intermedius*) fed different diets (mean ± SEM, n = 6) ^1^.

Index	Dietary Treatments						
	E78	E105	E152	E235	E302	E390	HD
Pepsin (U/mg prot)	6.42 ± 0.33 ^a^	6.75 ± 0.20 ^a^	5.90 ± 0.21 ^a^	7.00 ± 0.33 ^a^	7.42 ± 0.91 ^a^	3.91 ± 0.55 ^b^	7.13 ± 0.57 ^a^
Lipase (U/g prot)	5.99 ± 0.34 ^abc^	6.12 ± 0.19 ^abc^	6.23 ± 0.37 ^ab^	6.82 ± 0.20 ^a^	5.01 ± 0.44 ^cd^	4.63 ± 0.64 ^d^	5.58 ± 0.25 ^bcd^
Amylase (U/mg prot)	3.61 ± 0.50 ^bc^	4.54 ± 0.97 ^ab^	5.04 ± 0.59 ^ab^	5.87 ± 0.52 ^a^	3.77 ± 0.49 ^bc^	3.28 ± 0.23 ^bc^	2.72 ± 0.60 ^c^
Cellulase (U/mg prot)	18.83 ± 2.78 ^ab^	23.31 ± 0.48 ^a^	15.74 ± 0.96 ^bc^	15.79 ± 2.46 ^bc^	15.23 ± 1.84 ^bc^	11.35 ± 1.33 ^c^	22.13 ± 2.47 ^a^

^1^ Mean values with different superscript letters within the same row are significantly different at *p* < 0.05. Fresh kelp (HD).

**Table 5:** Immune- and antioxidation-related parameters in the coelomic fluid of sea urchin (*Strongylocentrotus intermedius*) fed different diets (mean ± SEM, n = 5) ^1^.

Index	Dietary Treatments						
	E78	E105	E152	E235	E302	E390	HD
LYZ (U/mL)	168.54 ± 29.00 ^a^	129.59 ± 16.93 ^a^	112.35 ± 1.98 ^ab^	163.29 ± 23.94 ^a^	154.49 ± 9.05 ^a^	155.06 ± 31.19 ^a^	57.98 ± 7.96 ^c^
AKP (U/100 mL)	1.27 ± 0.24 ^a^	0.81 ± 0.12 ^bc^	0.64 ± 0.12 ^bc^	0.99 ± 0.11 ^ab^	0.65 ± 0.31 ^bc^	0.66 ± 0.08 ^bc^	0.41 ± 0.03 ^c^
ACP (U/100 mL)	3.06 ± 0.36 ^a^	1.52 ± 0.18 ^bc^	1.07 ± 0.02 ^bc^	2.29 ± 0.13 ^ab^	1.96 ± 0.44 ^ab^	1.99 ± 0.62 ^ab^	0.64 ± 0.05 ^d^
SOD (U/mL)	57.27 ± 2.81 ^a^	47.96 ± 2.50 ^b^	47.46 ± 2.16 ^b^	53.85 ± 1.79 ^ab^	48.27 ± 1.11 ^b^	48.40 ± 2.11 ^b^	53.45 ± 0.50 ^ab^
CAT (U/mL)	0.80 ± 0.03 ^a^	0.51 ± 0.08 ^bc^	0.36 ± 0.00 ^d^	0.39 ± 0.05 ^cd^	0.62 ± 0.03 ^b^	0.55 ± 0.02 ^b^	0.63 ± 0.05 ^ab^
GST (U/mL)	12.70 ± 0.28 ^a^	8.61 ± 1.72 ^b^	3.12 ± 0.18 ^c^	7.33 ± 0.64 ^b^	7.64 ± 0.37 ^b^	8.85 ± 0.58 ^b^	11.92 ± 0.38 ^a^
GPX (U/mL)	24.74 ± 1.47 ^a^	19.71 ± 1.23 ^b^	14.89 ± 1.15 ^c^	21.60 ± 0.76 ^ab^	21.71 ± 0.39 ^ab^	22.79 ± 0.58 ^ab^	23.79 ± 0.52 ^a^
MDA (nmol/mL)	0.64 ± 0.09 ^b^	0.47 ± 0.02 ^bc^	0.61 ± 0.04 ^b^	0.90 ± 0.06 ^a^	0.53 ± 0.03 ^b^	0.55 ± 0.03 ^b^	0.33 ± 0.07 ^c^

^1^ Mean values with different superscript letters within the same row are significantly different at *p* < 0.05. Fresh kelp (HD), lysozyme (LYZ), alkaline phosphatase (AKP), acid phosphatase (ACP), superoxide dismutase (SOD), catalase (CAT), glutathione peroxidase (GPX), glutathione S-transferase (GST), and the content of malondialdehyde (MDA).

**Table 6:** Relative abundance of the intestinal microbiota at the phylum level of sea urchin (*Strongylocentrotus intermedius*) in response to different dietary treatments (mean ± SEM, n = 3) ^1^.

Phylum	Dietary Treatments			
	E78	E152	E390	HD
Proteobacteria	0.31 ± 0.01 ^b^	0.71 ± 0.03 ^a^	0.71 ± 0.05 ^a^	0.75 ± 0.01 ^a^
Firmicutes	0.32 ± 0.01 ^a^	0.08 ± 0.02 ^b^	0.07 ± 0.00 ^b^	0.09 ± 0.02 ^b^
Actinobacteria	0.10 ± 0.03 ^ab^	0.08 ± 0.02 ^ab^	0.12 ± 0.03 ^a^	0.06 ± 0.00 ^b^
Bacteroidetes	0.18 ± 0.01 ^a^	0.03 ± 0.01 ^b^	0.02 ± 0.00 ^b^	0.04 ± 0.00 ^b^
Firmicutes/Bacteroidetes	1.88 ± 0.18 ^b^	3.37 ± 0.20 ^ab^	4.14 ± 0.81 ^a^	2.48 ± 0.51 ^ab^

^1^ Mean values with different superscript letters within the same row are significantly different at *p* < 0.05. HD: fresh kelp.

**Table 7:** Relative abundance of the intestinal microbiota at the genus level of sea urchin (*Strongylocentrotus intermedius*) fed different diets (mean ± SEM, n = 3) ^1^.

Genus	Dietary Treatments			
	E78	E152	E390	HD
*Ruegeria*	0.02 ± 0.00 ^c^	0.35 ± 0.03 ^b^	0.42 ± 0.01 ^a^	0.47 ± 0.01 ^a^
*Phaeobacter*	0.01 ± 0.00 ^c^	0.14 ± 0.03 ^a^	0.08 ± 0.01 ^b^	0.09 ± 0.01 ^b^
*Rhodococcus*	0.07 ± 0.01 ^a^	0.05 ± 0.00 ^b^	0.07 ± 0.00 ^a^	0.04 ± 0.00 ^b^
*Maliponia*	0.00 ± 0.00 ^b^	0.08 ± 0.02 ^a^	0.12 ± 0.01 ^a^	0.02 ± 0.01 ^b^
*Escherichia-Shigella*	0.17 ± 0.01 ^a^	0.01 ± 0.00 ^b^	0.01 ± 0.00 ^b^	0.01 ± 0.00 ^b^
*Bacteroides*	0.12 ± 0.01 ^a^	0.01 ± 0.00 ^b^	0.01 ± 0.00 ^b^	0.01 ± 0.00 ^b^
*Clostridium sensu stricto* *1*	0.06 ± 0.00 ^a^	0.01 ± 0.00 ^b^	0.00 ± 0.00 ^b^	0.00 ± 0.00 ^b^

^1^ Mean values with different superscript letters within the same row are significantly different at *p* < 0.05. HD: fresh kelp.

## Data Availability

The data presented in this study are available on request from the corresponding author. The data are not publicly available due to privacy.
